# Impact of transient and chronic loneliness on progression and reversion of frailty in community-dwelling older adults: four-year follow-up

**DOI:** 10.1186/s12877-022-03283-1

**Published:** 2022-08-04

**Authors:** Bin-Lin Chu, Wen Zhang

**Affiliations:** 1 School of Nursing, Anqing Medical College, Anqing, China; 2 Department of Social Work and Social Administration, The University of Hong Kong, Pok Fu Lam, Hong Kong SAR, China

**Keywords:** Frailty, Loneliness, Older adults

## Abstract

**Background:**

Frailty is a common condition in older adults that is characterized by transitions between frailty states in both directions (progression and reversion) over time. Loneliness has been reported to be associated with the incidence of frailty, but few studies have explored the impact of persistent loneliness over time on frailty. In this study, we aimed to whether and how two different types of loneliness, transient and chronic, were associated with changes in frailty status in older adults.

**Methods:**

The analytic sample contained 2961 adults aged ≥ 60 years who completed interviews for both the 2011 and 2015 waves of the China Health and Retirement Longitudinal Study. The logistic regression model was used to examine the relationship between transient and chronic loneliness and progression and reversion of frailty. Demographics (age, sex, education level, marital status, urban–rural residence), living alone, chronic conditions, physical function, and depressive symptoms from the 2011 wave were adjusted.

**Results:**

After four years, 21% of the studied sample reported progression, 20% reported reversion in frailty, 31% reported transient loneliness, and 14% reported chronic loneliness. There was no significant difference in participants who reported transient loneliness (OR = 1.10, 95% CI [0.89,1.37]), or chronic loneliness (OR = 1.14, 95% CI [0.84,1.57]) on the progression of frailty, compared with no report of loneliness. Participants reporting chronic loneliness (OR = 0.68, 95% CI [0.50,0.93]) were less likely to report reversion in their level of frailty compared to participants who did not report loneliness but not transient loneliness (OR = 0.87, 95% CI [0.70,1.08]).

**Conclusions:**

Roughly the same percentage, a fifth, of older Chinese adults progressed or reversed in frailty status without active intervention. Chronic loneliness was related to a lower probability of reversion in the frail group than in the no loneliness group, but not in the transient loneliness group. More attention should be given to older adults with chronic loneliness.

**Supplementary Information:**

The online version contains supplementary material available at 10.1186/s12877-022-03283-1.

## Introduction

Frailty is a common condition in older adults that characterizes their susceptibility to poor homeostasis resolution after an abrupt change in health status. According to Fried’s phenotype model [1], frailty is the presence of at least three of five criteria: slow gait speed, inactivity, weakness (grip strength), self-reported exhaustion, and unintentional weight loss. In this model, frailty status was categorized as robustness(or nonfrailty), prefrailty, and frailty. Frailty will become more prevalent as the population ages, putting a greater strain on health and well-being. This in turn will have a significant impact on health and social care resources and result in a huge burden on clinical practice and public health [2]. As a result, it is crucial to determine how to slow the onset of frailty.

The frailty process is not a steady state but is characterized by transitions between frailty states in both directions (progression and reversion) over time [3]. A meta-study pooled data from 42,775 community-dwelling older adults and showed that transitions between adjacent states (one-step transitions) are prevalent [4, 5]. Frailty transitions have been demonstrated to be influenced by a variety of health and psychosocial variables, such as loneliness in recent research [6].

Loneliness is a subjective distressing feeling generated by an individual’s impression of a gap between their actual and desired social relationships [7]. Although loneliness may affect people of any age, it has a particularly negative influence on older adults [8]. A recent study systemtatically reviewed 57 studies from 113 countries or territories and reported a 21% to 24% of prevalence of loneliness in older adults, which was approximately 10 times that in young adults [9]. Increasing evidence indicates that the onset of the COVID-19 pandemic, along with the constainment measures — such as social distancing restrictions; the ‘stay at home’ order; and the closure of cafes, restaurants, and gyms — have disproportionately affected older adults, increasing the incidence of loneliness [10]. Loneliness is influenced by external circumstances (for example, bereavement and migration) and the human characteristics of the individual such as health status and personality [11]. As a result, the level of loneliness experienced by older adults is likely to change over time (referred to here as “transient” loneliness) in some people, while it remains constant in others (referred to here as “chronic” loneliness) [12].

An increasing number of studies have explored the association between loneliness and frailty in older adults. In general, older adults with frailty appear to have fewer social networks and greater rates of loneliness [13]. In return, loneliness is also a risk factor for the incidence of frailty [14]. To date, most research has concentrated on the association between frailty progression and severe/regular loneliness [6, 15]. Few concern the impact of persistent loneliness over time. Only a four-year cohort study found that older adults with chronic loneliness had a higher risk of death than those with transient loneliness [16]. However, it is unclear whether transient and chronic loneliness have distinct effects on older adults’ frailty status changes. Hence, in this study, we aimed to identify the prevalence and explore the potentially different impacts of transient and chronic loneliness on the progression and reversion of frailty by using data from the China Health and Retirement Longitudinal Study (CHARLS).

## Methods

### Data and sample

Data were obtained from the ongoing CHARLS. For geriatric and health policy research, the CHARLS gathered a nationwide representative cohort of Chinese residents aged over 45 and older to collect a wide range of personal health information as well as social and economic data. Approximately 150 regions (counties) were chosen according to population size from 28 Chinese provinces, and three villages/communities were then selected from each county as primary sample units (PSUs). Each of the 450 PSUs had eighty households picked at random, with 24 being investigated. If there were people in the family who were 45 or older, one of them was chosen at random as a respondent, and both the respondents and their spouses were interviewed. There are currently four waves of data available, including at baseline (wave 1 in 2011) and follow-ups every two years at wave 2 (2013), wave 3 (2015) and wave 4 (2018). At the baseline of the study, a total of 17,708 people from 10,257 households were enrolled. The details of the cohort have been described elsewhere [17]. The CHARLS was approved by the ethical review committee at Peking University. The participants in the CHARLS were interviewed face-to-face using a standardized questionnaire.

**Fig. 1:**
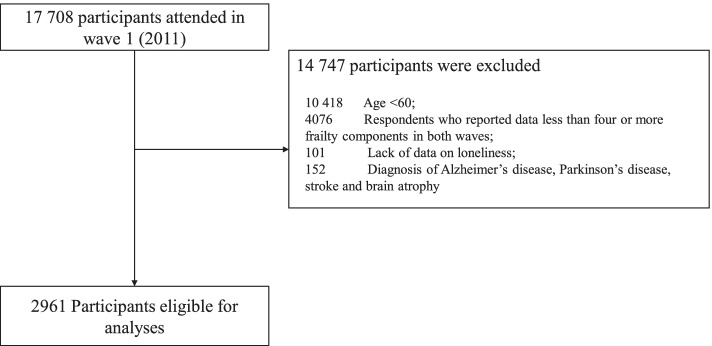
Flow chart showing the selection of the participants in this study

Since the measurement of frailty was lacking in 2018, the data from 2011 and 2015 included in this study to obtain the longest follow-up. The inclusion criteria of this study’s cohort were as follows: (1) adults aged 60 and above in 2011 and (2) respondents who reported data on four or more frailty components in 2011 and 2015. The exclusion criteria were as follows: (1) no data on loneliness and (2) diagnosis of Alzheimer’s disease, Parkinson’s disease, stroke or brain atrophy. Ultimately, the total sample size was 2961, and a detailed flow chart is shown in Fig. 1.

### Measures

#### Dependent variables: progression and reversion of frailty

Frailty was measured with the modified Frailty Phenotype Criteria (PFC) which have been rigorously validated using CHARLS data [1]. If three or more of the following five criteria are met, older adults were identified as frail (coded “2”): slow walking speed, weakness, exhaustion, shrinking, and inactivity. Older adults who met one or two criteria were identified as prefrail (coded “1”), and those who met none were coded 0 for robust. There were two binary variables to identify progression cases and reversion cases separately. Individuals who were initially robust or prefrail (in 2011) and progressed to severe status (prefrail or frail) in 2015 were identified as cases of “progression” (coded “1”), while others were coded “0” in the “progression” variable. Individuals who were initially prefrail or frail and reversed to mild status (prefrail or robust) were identified as cases of “reversion” (coded “1”), while others were coded “0” in the “reversion” variable.

*Slow walking* speed was defined as the average time of two walking tests on a 2.5-m track that was slower than the 20% percentile adjusted for sex and height. *Weakness* was defined as the maximal grip strength of each hand (two tests), assessed by a Yuejian™ WL-1000 mechanical dynamometer, which was lower than the 20th percentile adjusted for sex and BMI. *Exhaustion* was identified if the participants reported a moderate amount of time or all of the time to either of two questions (“I could not get moving” and “I felt like everything I did was an effort”). *Shrinking* was defined as self-reported weight reduction of 5 kg or more in the previous year or a current BMI of less than 18.5 kg/m^2^. *Inactivity* was identified by an item asking individuals if they walked for 10 min or more constantly throughout a typical week.

#### Independent variables: loneliness

Loneliness was measured with a single item (how often did you feel lonely) of the Centre for Epidemiological Studies Depression Scale (CESD) in 2011 and 2015. Responses were scored on a 4-point Likert scale from 0 (never) to 3 (always). Following the previous study [18], individuals responding “some of the time”, “occasionally”, or “always” were recorded as “lonely”. This measurement has been widely used in the Chinese population [19]. Following previous research [20], transient loneliness was defined if individuals reported “lonely” in one of waves, and chronic loneliness if they reported “lonely” in both waves and not lonely if they reported not lonely in both waves.

#### Covariates

Age, sex, education level, marital status, current residence location, living alone, chronic conditions, physical function (activities of daily living (ADLs) and instrumental activities of daily living (IADLs)), and depressive symptoms were adjusted as covariates. There were three levels of educational attainment, including no formal education, elementary or middle school, and high school or above. Marital status was categorized as “married/partnered” or “unmarried”. The current resident location was categorized as residing in a rural area or an urban area. A chronic condition was considered present if the participant had been diagnosed with one or more of the following chronic diseases: diabetes, cancer, heart attack, chronic lung diseases, and liver disease. Physical function was assessed by ADLs and IADLs. ADLs were considered present if the older adults had difficulties in eating, bathing, dressing, controlling their urine, using the toilet, or getting in and out of bed with a range of 0–6. The IADLs with a range of 0–6 were measured if older adults had difficulties with housework, preparing hot meals, grocery shopping, making phone calls, taking medication, or manging money. Depressive symptoms were measured by the modified Chinese version of the CES-D [21] excluding two items for identifying exhaustion (in the frailty) and one item for measuring loneliness with a range of 0–21.

### Statistical analysis

The demographic characteristics were summarized by using descriptive statistics. Logistic regression models were used to examine the impact of independent variables either on the progression or reversion of frailty status. A low proportion (2.7%) of respondents had at least 1 missing value on covariates, and those respondents were excluded from the Logistic regression model analysis. Multiple imputation was applied in the sensitivity analysis. A total of 30 imputed data set were obtained and the results were pooled by Rubin’s rule [22]. The consistent results were found and details of the sensitivity analysis are shown in the Supplementary Information. The hazard ratio (HR) and corresponding 95% confidence intervals (95% CI) were reported. Statistical analyses were conducted using R version 4.0.4.

## Results

Table 1 presents the descriptive chracteristics for the whole sample differentiated by frailty status (in 2011). Participants had an average age of 67 at the baseline; 49% were female, a majority had no formal education (55.3%), 81.6% were married, and 31.9% lived in urban areas. Among them, 967 (32.7%) were robust, 1848 (62.4%) were pre-frail, and 146 (4.9%) were frail.

**Table 1:** Sample Characteristics and progression and reversion rate

	All participates	Robust (in 2011)	Pre-frail (in 2011)	Frail (in 2011)
Demographic characteristics (Mean ± SD / N(%))	*N* = 2961	*N* = 967	*N* = 1848	*N* = 146
Age (Mean ± SD)	67 ± 5.52	65 ± 4.82	67 ± 5.57	72 ± 6.01
Sex				
Male	1510(51.0%)	549(56.8%)	903(48.9%)	58(39.7%)
Female	1451(49.0%)	418(43.2%)	945(51.1%)	88(60.3%)
Educational Level				
No formal education	1638(55.3%)	472(48.8%)	1061(57.4%)	105(71.9%)
Elementary school and middle school	1181(39.9%)	427(44.2%)	715(38.7%)	39(26.7%)
High school and higher	142(4.8%)	68(7.0%)	72(3.9%)	2(1.4%)
Marital status				
Married/partnered	2417(81.6%)	822(85.0%)	1496(81.0%)	99(67.8%)
Unmarried	544(18.4%)	145(15.0%)	352(19.0%)	47(32.2%)
Current Residential location				
Urban	945(31.9%)	359(37.1%)	549(29.7%)	37(25.3%)
Rural	2016(68.1%)	608(62.9%)	1299(70.3%)	109(74.7%)
Number of chronic conditions (0 ~ 9)	1 ± 1.37	1 ± 1.27	2 ± 1.40	2 ± 1.42
Missing	80(2.7%)	28(2.8%)	45(2.4%)	7(4.7%)
Living alone				
No	2727(92.1%)	902(93.3%)	1701(92.0%)	124(84.9%)
Yes	234(7.9%)	65(6.7%)	147(8.0%)	22(15.1%)
Depression(0-)	8.76 ± 6.23	5.37 ± 3.82	10.14 ± 6.50	13.84 ± 5.59
ADLs(0–6)	0.42 ± 0.93	0.14 ± 0.49	0.43 ± 0.99	0.81 ± 1.41
Missing	17(0.6%)	2(0.2%)	13(0.7%)	2(1.3%)
IADLs(0–5)	0.36 ± 0.90	0.23 ± 0.65	0.47 ± 0.98	1.04 ± 1.40
Missing	24(0.8%)	8(0.8%)	16(0.8%)	0(0%)

ADLs referred to Activities of Daily Living (ADLs) and IADLs referred to Instrumental activities of daily living (IADLs)

**Fig. 2:**
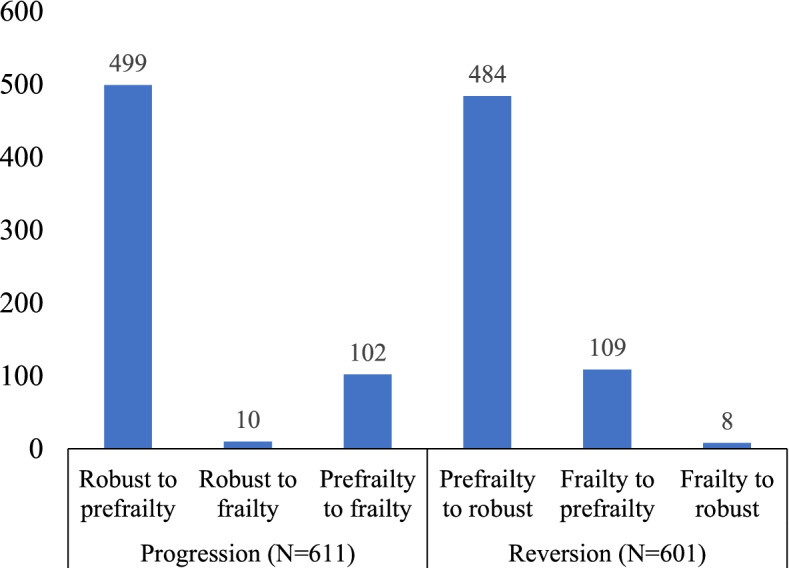
Distribution of progression and reversion

After four years, 21% (611/2961) of subjects reported progression, and 20% (601/2961) reported reversion of frailty. The distribution of progression and reversion according was shown in Fig. 2. Among participants reported progression, 82% (499/601) were progressed from robust to prefrailty while 16% (102/601) were progressed from prefrailty to frailty and 2% (10/601) from robust to frailty. Among participants reported reversion, 81% (484/601) were reversed from prefrailty to robust while 18% (109/601) were reversed from frailty to prefrailty and 1% (8/601) from frailty to robust.

**Fig. 3:**
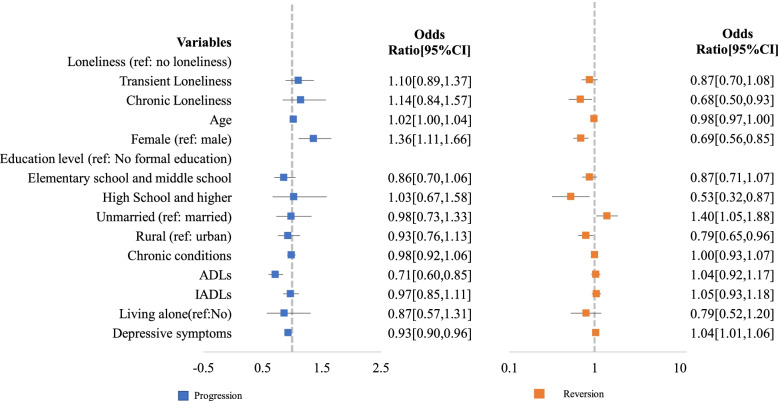
Results for the adjusted Cox Proportional Hazards Model of Progression and Reversion of Frailty. Note: Models were adjusted by age, sex, education level, marital status, current residence location, the presence of the chronic condition, ADLs, IADLs, living alone, and depressive symptoms. ADLs refer to Activities of Daily Living (ADLs) and IADLs refer to Instrumental activities of daily living (IADLs)

After four years, 31% (927/2961) reported transient loneliness, and 14% (426/2961) reported chronic loneliness. The results of the adjusted logistic regression model regarding progression and reversion are shown in Fig. 3. There was no significant difference in participants who reported transient loneliness (OR = 1.10, 95% CI [0.89,1.37]) or chronic loneliness (OR = 1.14, 95% CI [0.84,1.57]) on the progression of frailty, compared with no loneliness. Participants reporting chronic loneliness (OR = 0.68, 95% CI [0.50,0.93]) were less likely to report reversion in their level of frailty compared to participants who did not report loneliness, while there was no significant difference in transient loneliness (OR = 0.87, 95% CI [0.70,1.08]) and no loneliness.

## Discussion

To our best knowledge, this study was the first to investigate the association between different types of loneliness and progression and reversion of frailty in older Chinese adults. The findings from this representative Chinese community-dwelling sample indicated that approximately one-fifth of the sample progressed their frailty status and the roughly same percentage of older adults reversed their status, which is similar to a previous study in Europe [6]. Although some older adults reported reversion without intervention, a majority of older adults remained the same status or reported progression when the participants had already been frail or prefrail. It means that the early intervention is still warranted to prevent or reduce the level of frailty in older adults.

We found that chronic loneliness was related to a lower probability of reversion in the frail group than in the no loneliness group but not in the transient loneliness group. These findings highlight that chronic loneliness may act as an obstacle to spontaneous reversion. This might be because people with loneliness may have higher immune functioning, such as higher levels of tumor necrosis factor alpha (TNF-α) and interleukin-6 (IL-6) [23], which have also been found to be high in frail older adults [24]. This effect may require accumulation [25], which may lead to no significant effect on the transient loneliness group. Another potential mechanism is that chronic loneliness may provide a further obstacle to successfully modifying social networks in accordance with individuals’ abilities and may then contribute to a series of physical and mental health conditions, including stroke, chronic stress, depression, and cognitive decline [26], all of which may decrease the likelihood of spontaneous reversion. Our findings further underlying that there is a difference in the effects of subtypes of loneliness. On the other hand, we also found that both transient loneliness and chronic loneliness had no association with the progression of frailty, in contrast to a previous study conducted by Gale and colleagues [15]. This may be because Gale’s study only measured the level of loneliness at the baseline instead of a dynamic condition measured in our study. A further systematic review of the association between the two subtypes of loneliness and frailty might be warranted.

Our findings have some key implications for the management of frailty. First, our study provides an overview of the frailty transition among older adults in China. Although the frailty in older adults could be spontaneously reversed, a majority of older adults remained the same status or reported progression when the participants had already been frail or prefrail. Early intervention for older adults is still important. Second, our study highlights that chronic loneliness may prevent reversion in those who might otherwise improve their frailty status. Although health and social care providers place a strong premium on diet and physical activity, social interaction elements, such as loneliness, have received less attention. Our study identifies a vulnerable group, chronically lonely older adults, and suggests that this vulnerable group is more likely to be helped by early interventions to increase the probability of reversing frailty, which might reduce the healthcare burden for older adults.

The strengths of our study include the use of a large sample size and long-term follow-up. This study also explored the differential impact of transient and chronic loneliness on changes in frailty status. There are some limitations to interpreting the results. First, our sample only included respondents who had reported in 2011 and 2015 and a number of subjects were excluded because of death or being lost to follow-up. Selection bias might occur since participants included in the analysis were more likely to be healthier than those who were excluded. Second, loneliness was assessed by one item, although this measurement has been widely used in the Chinese population [19] and has strong correlations with multiple items. This single-item measurement may be less reliable than a measure with multiple aspects of loneliness [27]. Third, loneliness was measured in a relatively short period. In this study, participants reported their psychosocial condition in the last week, which may not reflect the fluctuation during the 4-year follow-up period. A long-term measurement of loneliness might be warranted in future studies.

In conclusion, one-fifth of older Chinese adults progressed, and the roughly same percentage of older adults reversed their frailty status. Chronic loneliness was related to a lower probability of reversion in the frail group than in the no loneliness group but not in the transient loneliness group. Attention should be given to older adults with constant loneliness, which should be addressed.

## Supplementary Information

**Additional file 1: Table1.** Adjusted Cox Proportional-Hazards Model of Progression and Reversion ofFrailty After Multiple Imputation.

## Data Availability

The datasets used in the current study are accessed in the CHARLS website (http://forum.charls.pku.edu.cn/) after the users apply in their website.

## Abbreviations

ADLs: Activities of Daily Living
CES-D: Epidemiological Studies-Depression
CHARLS: China Health and Retirement Longitudinal Study
HR: Hazard Ratio
IADLs: Instrumental Activities of Daily Living
IL-6: Interleukin-6
PFC: Frailty Phenotype Criteria
PSUs: Primary Sample Units
TNF- α: Tumor Necrosis Factor Alpha
