# Supplementary material for: Impact of transient and chronic loneliness on progression and reversion of frailty in community-dwelling older adults: four-year follow-up
Source: BMC Geriatr. 2022 Aug 4;22:642. doi: 10.1186/s12877-022-03283-1 (PMC9351253; doi:10.1186/s12877-022-03283-1)
Supplement: Supplementary file 1 — Additional file 1: Table1. Adjusted Cox Proportional-Hazards Model of Progression and Reversion ofFrailty After Multiple Imputation. [file 12877_2022_3283_MOESM1_ESM.docx]

**Supplementary Information**

**Sensitivity Analysis**

Table 1 Adjusted Cox Proportional-Hazards Model of Progression and Reversion of Frailty After Multiple Imputation

|  | Progression | | | Reversion | |
| --- | --- | --- | --- | --- | --- |
|  | Odds Ratio | 95%CI | Odds  Ratio | | 95%CI |
| Transient Loneliness | 1.09 | [0.88,1.35] | 0.89 | | [0.72,1.10] |
| Chronic Loneliness | 1.13 | [0.83,1.53] | 0.71* | | [0.52,0.96] |
| Adjust variables |  |  |  | |  |
| Age | 1.02* | [1.002,1.04] | 0.99 | | [0.97,1.003] |
| Sex (ref: male) |  |  |  | |  |
| Female | 1.37** | [1.12,1.67] | 0.69*** | | [0.57,0.84] |
| Education level (ref: No formal education) |  |  |  | |  |
| Elementary school and middle school | 0.87 | [0.71,1.07] | 0.85 | | [0.69,1.04] |
| High School and higher | 1.04 | [0.68,1.60] | 0.54* | | [0.33,0.88] |
| Marital status (ref: married) |  |  |  | |  |
| Unmarried | 0.99 | [0.74,1.33] | 1.33 | | [1.00,1.77] |
| Current residence location (ref: urban) |  |  |  | |  |
| Rural | 0.94 | [0.77,1.15] | 0.76** | | [0.63,0.92] |
| Chronic conditions | 0.99 | [0.92,1.06] | 1.00 | | [0.93,1.07] |
| ADLs | 0.73*** | [0.62,0.86] | 1.05 | | [0.93,1.18] |
| IADLs | 1.00 | [0.88,1.14] | 1.03 | | [0.92,1.16] |
| Living along(ref:No) |  |  |  | |  |
| Yes | 0.87 | [0.58,1.31] | 0.84 | | [0.56,1.27] |
| Depressive symptoms | 0.93*** | [0.90,0.96] | 1.04** | | [1.01,1.06] |

Note: Models were adjusted by age, sex, education level, marital status, current residence location, memory problem, the presence of the chronic condition, frailty status, living alone, and depressive symptoms (wave 1).

*<0.05, **<0.01, ***<0.001
